# Phosphorylation of PLK3 Is Controlled by Protein Phosphatase 6

**DOI:** 10.3390/cells9061506

**Published:** 2020-06-20

**Authors:** Cecilia Aquino Perez, Matous Palek, Lenka Stolarova, Patrick von Morgen, Libor Macurek

**Affiliations:** Cancer Cell Biology, Institute of Molecular Genetics of the Czech Academy of Sciences, CZ14220 Prague, Czech Republic; Cecilia.Aquino-Perez@img.cas.cz (C.A.P.); matous.palek@img.cas.cz (M.P.); Lenka.Stolarova@img.cas.cz (L.S.); vonmorgenster@gmail.com (P.v.M.)

**Keywords:** protein kinase, Polo-like kinase 3, protein phosphatase, stress, DNA damage

## Abstract

Polo-like kinases play essential roles in cell cycle control and mitosis. In contrast to other members of this kinase family, PLK3 has been reported to be activated upon cellular stress including DNA damage, hypoxia and osmotic stress. Here we knocked out *PLK3* in human non-transformed RPE cells using CRISPR/Cas9-mediated gene editing. Surprisingly, we find that loss of PLK3 does not impair stabilization of HIF1α after hypoxia, phosphorylation of the c-Jun after osmotic stress and dynamics of DNA damage response after exposure to ionizing radiation. Similarly, RNAi-mediated depletion of PLK3 did not impair stress response in human transformed cell lines. Exposure of cells to various forms of stress also did not affect kinase activity of purified EGFP-PLK3. We conclude that PLK3 is largely dispensable for stress response in human cells. Using mass spectrometry, we identify protein phosphatase 6 as a new interacting partner of PLK3. Polo box domain of PLK3 mediates the interaction with the PP6 complex. Finally, we find that PLK3 is phosphorylated at Thr219 in the T-loop and that PP6 constantly dephosphorylates this residue. However, in contrast to PLK1, phosphorylation of Thr219 does not upregulate enzymatic activity of PLK3, suggesting that activation of both kinases is regulated by distinct mechanisms.

## 1. Introduction

Polo-like kinases (Plks) are evolutionary conserved Ser/Thr protein kinases that play critical roles in progression through the cell cycle and mitosis [1]. All Plks share similar structures with N-terminal catalytic domain and two or more C-terminal polo boxes that serve as a substrate-binding domain [2]. In vertebrates, Polo-like kinase family comprises of five members including PLK1 that is essential for formation of a bipolar mitotic spindle and for cytokinesis [3,4]; PLK2 and PLK4 that are involved in centriole biogenesis and duplication [5]; and PLK5 that lacks the kinase activity and plays a structural role in neurons [6]. Out of the Plks, the function of PLK3 is the least explored. Originally, human PLK3 (previously also reported as FGF-inducible kinase (Fnk)) was found to localize to plasma membrane where it was shown to modulate cell adhesion in specialized cell types including macrophages [7,8]. PLK3 was also implicated in the cell cycle, in particular in control of G1/S and G2/M transitions through promoting nuclear translocation of CDC25A and CDC25C, respectively [9,10]. However, the function of PLK3 in the cell cycle is likely not essential as PLK3 knock-out mice are viable and fertile [11]. PLK3 was suggested to control cell response to various forms of stress including osmotic stress, hypoxia, DNA damage, and Golgi stress [12]. Whereas PLK1 is rapidly inhibited after stress, activity of PLK3 is believed to be stimulated by stress [10,12,13]. Following DNA damage, PLK3 is supposed to phosphorylate CHK2 at Ser62 and p53 at Ser20, leading to activation of the cell cycle checkpoint [13,14]. In addition, PLK3 was shown to promote DNA repair in G1 cells by phosphorylating a chromatin-bound C-terminal binding protein–interacting protein (CtIP) [15]. Hypoxia or hypoxia-mimicking treatment with CoCl2 was reported to activate PLK3 in the nucleus and to negatively regulate HIF1α levels in murine cells [16,17,18]. Upon hyperosmotic stress, PLK3 was reported to phosphorylate c-Jun and γH2AX in human corneal epithelia [19,20]. Finally, PLK3 was shown to be activated upon and contribute to Golgi fragmentation induced by nocodazole or brefeldin A treatment [21,22,23].

Serine/threonine protein phosphatase 6 (PP6) is a heterotrimeric complex consisting of the catalytic subunit (PPP6C), SAPS domain-containing subunit (PPP6R1-3) and ankyrin repeat-domain containing regulatory subunit (ANKRD28, 44, 52) [24]. PP6 was implicated in cell cycle progression, DNA damage repair, inflammatory signaling, and lymphocyte development [25]. During mitosis, PP6 dephosphorylates Aurora-A and depletion of PP6 impairs mitotic spindle formation [26,27]. In addition, PP6 interacts with and controls the activation of DNA-PK after DNA damage and modulates the sensitivity of glioblastoma to ionizing radiation [28,29]. PP6 is commonly mutated in melanoma and loss of PP6 promoted skin cancerogenesis induced by DMBA [30,31].

Here we used CRISPR/Cas9-mediated gene editing and inactivated PLK3 in human non-transformed cells to study the involvement of PLK3 in the cellular response to stress. Surprisingly we find that PLK3 plays redundant roles in cell response to DNA damage, osmotic stress and hypoxia. In agreement with these findings, we did not observe significant changes in the kinase activity of PLK3 purified from cells exposed to various forms of stress. To search for protein interactors that could modulate the function of PLK3, we used mass spectrometry and identified PP6 holoenzyme in a stable complex with PLK3. We find that similarly to PLK1, PLK3 is also phosphorylated in the T-loop and that inhibition of protein phosphatases increases the level of PLK3 modification. However, we found that mutation of the Thr219 in the T-loop did not affect the activity of PLK3, suggesting that the mechanism of its regulation is distinct to PLK1.

## 2. Materials and Methods

### 2.1. Antibodies and Reagents

The following antibodies were used: rabbit monoclonal to PLK3 (clone D14F12, Cell Signaling Technology, #4896); rabbit polyclonal to PLK3 (Novus Biologicals, Abingdon, UK, NBP23-2530); goat polyclonal to PLK3 (Bio-Rad, Hercules, CA, USA, VPA00063); rabbit polyclonal to PLK3 (Sigma-Aldrich, St. Louis, MO, USA, HPA060318); mouse monoclonal to PLK3 (clone B37-2, BD Pharmingen, San Jose, CA, USA, 556518); rabbit polyclonal to PLK3 (St. Johns Laboratory, London, UK, STJ93099); rabbit monoclonal Phospho-PLK1 (Thr210) Antibody (Cell Signaling Technology, Danvers, MA, USA #9062); γ-tubulin (Sigma-Aldrich); GM130, p38-pT180/pY182, cJun-pSer73, phospho-histone H2AX-Ser139 (referred to as γ-H2AX) and HIF1α (Cell Signaling Technology); PPP6R1, PPP6R2 and PPP6R3 (A300-968A, A300-970A, A300-972A, Bethyl Laboratories, Montgomery, TX, USA); 14-3-3, mouse monoclonal to PPP6C and TFIIH (Santa Cruz Biotechnology, Dallas, TX, USA); and rabbit polyclonal to PPP6C (Abcam, Cambridge, UK). HRP-conjugated secondary antibodies were from Bio-Rad, Alexa Fluor-conjugates from ThermoFisher Scientific (Waltham, MA, USA). Etoposide, leptomycin B and nocodazole were from Sigma-Aldrich, calyculin from Santa Cruz Biotechnology.

### 2.2. Cells

Human hTERT-immortalized RPE1 cells (hereafter referred to as RPE) were obtained from ATCC and were grown in DMEM supplemented with 6% FBS (Gibco, Waltham, MA, USA), Penicillin and Streptomycin. Cells were regularly tested for mycoplasma infection using MycoAlert kit (Lonza, Basel, Switzerland). To generate PLK3 knock out cell line, RPE cells grown at 6-well plate were transfected with synthetic sgRNA (CRISPRevolution sgRNA EZ Kit; Synthego, Menlo Park, CA, USA) and recombinant EnGen Spy Cas9 NLS (New England Biolabs, Ipswich, MA, USA) using CRISPRMAX reagent (ThermoFisher Scientific). Two independent targeting sequences in exon 2 of human PLK3 were UGUCAGUGGCCUCGUAGCAG and GGGCUUCGCCCGCUGCUACG. Three days after transfection, single cells were seeded on 96-well plates and individual clones were expanded. Genomic DNA was isolated from individual clones and the fragment corresponding to DNA from intron 1 to exon 3 was amplified by PCR, sequenced and analyzed by TIDE software (Desktop Genetics, Cambridge, MA, USA). For selected clones, PCR fragments were inserted into pcr2.1-TOPO plasmid, and plasmid DNA from 10 bacterial colonies was sequenced to confirm individual alleles of *PLK3*. The following two independent clones were selected for further functional testing: RPE-PLK3-KO clone cr1.2 carries a single nucleotide insertion in the target site and a 54 bp deletion at intron/exon2 transition; RPE-PLK3-KO clone cr2.3 is a homozygote carrying a single nucleotide insertion within the target sequence in exon 2. Loss of PLK3 expression in the knock-out cells was further validated by immunoblotting. Silencer Select siRNA targeting PLK3 (PLK3 siRNA1 GGCUUUGGGUAUCAACUGU and PLK3 siRNA2 GCAUCAAGCAGGUUCACUA) was transfected at a final concentration of 5 nM using RNAiMAX (ThermoFisher Scientific) and cells were collected 48 h after transfection. HEK293 cells were transfected with pcDNA4/TO/EGFP-PLK3-myc plasmid and stable clones were selected by treating cells with zeocin for 3 weeks. Where indicated, cells were grown for 12 h in media supplemented with 150–300 μM CoCl2 to mimic hypoxia [16]. Hyperosmotic shock was induced by incubation of cells with media supplemented with 350 mM NaCl or 480 mM mannitol for 40 min [20]. Hypotonic shock was induced by incubation of cells with media diluted 1:1 with water.

### 2.3. Plasmids

pcDNA4/TO/PLK3-myc construct was obtained from Abgent. DNA fragment coding for EGFP was cloned in-frame into HindIII-EcoRI sites of pcDNA4/TO/PLK3-myc-His. Alternatively, fragment carrying PLK3 was subcloned into pEGFP-C2 (Clontech, Mountain View, CA, USA). PLK3-H590A-K592M, PLK3-K91R, PLK3-T219A, and PLK3-T219D mutants were generated by site-directed mutagenesis. To generate EGFP-PLK3-∆PBD mutant lacking the C-terminal PBD domain, we PCR amplified the fragment corresponding to amino acids 1–465 of PLK3 and ligated it into pcDNA4/TO/EGFP-myc backbone using Gibson assembly. All constructs were validated by sequencing.

### 2.4. Real-Time Quantitative Reverse Transcriptase PCR (qRT-PCR)

Total RNA was isolated 48 h after transfection of RPE cells with control or PLK3 siRNA using RNeasy Mini Kit (Quiagen, Hilden, Germany). cDNA was synthesized from 3 µg total RNAs using random hexamer and RevertAid H Minus Reverse Transcriptase (ThermoFisher Scientific). Real-time quantitative PCR was performed on LightCycler 480 Instrument II (Roche, Basel, Switzerland) using LightCycler 480 SYBR Green I Master (Roche) and the following primers: PLK3-forward TGAGGACGCTGACAACATCTAC, PLK3-reverse CAGGTAGTAGCGCACTTCTGG, ATP5B-forward TGAAGAA-GCTGTGGCAAAAGC, and ATP5B-reverse GAAGCTTTTTGGGTTAGGGGC. Relative amount of *PLK3* mRNA is presented as the ratio to *ATP5B* mRNA.

### 2.5. Immunofluorescence

Cells grown on coverslips were fixed with 4% paraformaldehyde for 15 min at room temperature and permeabilized with 0.5% Triton X1−00 for 10 min. Cells were further incubated with ice-cold methanol for 5 min and blocked with 3% BSA in PBS for 30 min. Coverslips were incubated with primary antibodies for 3 h, washed with PBS, and incubated with AlexaFluor-conjugated secondary antibodies for 1 h. Mounting was performed using Vectashield. Imaging was performed using Leica Sp8 confocal microscope equipped with 63× oil objective (NA 1.40). Images were analyzed using LAS AF Lite software (Leica, Wetzlar, Germany). Induction of DNA damage response was evaluated as described previously [32]. Briefly, cells were exposed to ionizing radiation (3 Gy) using X-RAD 225XL instrument (Precision; Cu filter 0.5 mm), fixed with 4% PFA, permeabilized with 0.5% Triton X1−00, and probed with antibody against γH2AX (Cell Signaling Technology). Images were acquired using Olympus ScanR system equipped with 40×/NA 1.3 objective (Olympus, Tokio, JApan). Number of γH2AX-positive foci per nucleus was determined using spot detection module. More than 300 nuclei were quantified per condition.

### 2.6. Immunoprecipitation

HEK293 cells stably expressing EGFP or EGFP-PLK3 were extracted by IP buffer (20 mM HEPES pH 7.5, 10% glycerol, 150 mM NaCl, 0.5% NP40) supplemented with cOmplete protease and PhosSTOP phosphatase inhibitors (Sigma) and sonicated for 3 × 20 s on ice. Cell extracts were cleared by centrifugation at 15,000 rpm 10 min at 4 °C and incubated with GFP-Trap beads (Chromotek, Planegg, Germany) for 2 h. After three washes in IP buffer, bound proteins were eluted from beads by Laemli buffer and analyzed by immunoblotting. Alternatively, bound proteins were analyzed by mass spectrometry using Orbitrap Fusion (Thermo Scientific). Proteins bound to EGFP-PLK3 that were enriched compared to the empty EGFP control in at least two out of three independent experiments were considered as potential interactors and were validated by immunoprecipitation followed by immunoblotting. For in vitro kinase assay, wild-type or mutant EGFP-PLK3 was immunoprecipitated using GFP Trap, washed three times in IP buffer and incubated with casein in kinase buffer (10 mM HEPES pH 7.4, 5 mM MgCl_2_, 2 mM EGTA, 1 mM DTT, 2.5 mM β-glycerolphosphate, 100 µM ATP and 5 µ Ci ^32^P-γ-ATP) for 20 min at 30 °C. Proteins were separated using SDS-PAGE, and phosphorylation was visualized by autoradiography.

### 2.7. Cell Fractionation

RPE cells were fractionated as described before [33,34]. Briefly, soluble cytosolic fraction was obtained by incubating cells in buffer A [10 mM HEPES pH 7.9, 10 mM KCl, 1.5 mM MgCl_2_, 0.34 M sucrose, 10% glycerol, 1 mM DTT, 0.05% Triton X1−00 and protease inhibitor cocktail] at 4 °C for 10 min and spinning down at 1500× *g* for 2 min. Pelleted nuclei were further extracted with an equal amount of buffer B [10 mM HEPES pH 7.9, 3 mM EDTA, 0.2 mM EGTA, 1 mM DTT] and spinning down at 2000× *g* for 2 min yielding a soluble nuclear fraction. Insoluble chromatin was washed with buffer B and resuspended in SDS sample buffer.

### 2.8. Statistical Analysis

Signal intensity of the bands in Western blots was measured from biological replicates (*n* ≥ 3) using gel analysis plug-in in ImageJ. After background subtraction, signal was normalized to the corresponding loading control and to non-treated condition. Statistical significance was evaluated using two-tailed Student’s T-test in Prism 5 software (GraphPad). Values *p* < 0.05 were considered statistically significant.

## 3. Results

### 3.1. PLK3 Localizes to Plasma Membrane, Golgi and Centrosome

**Figure 1:**
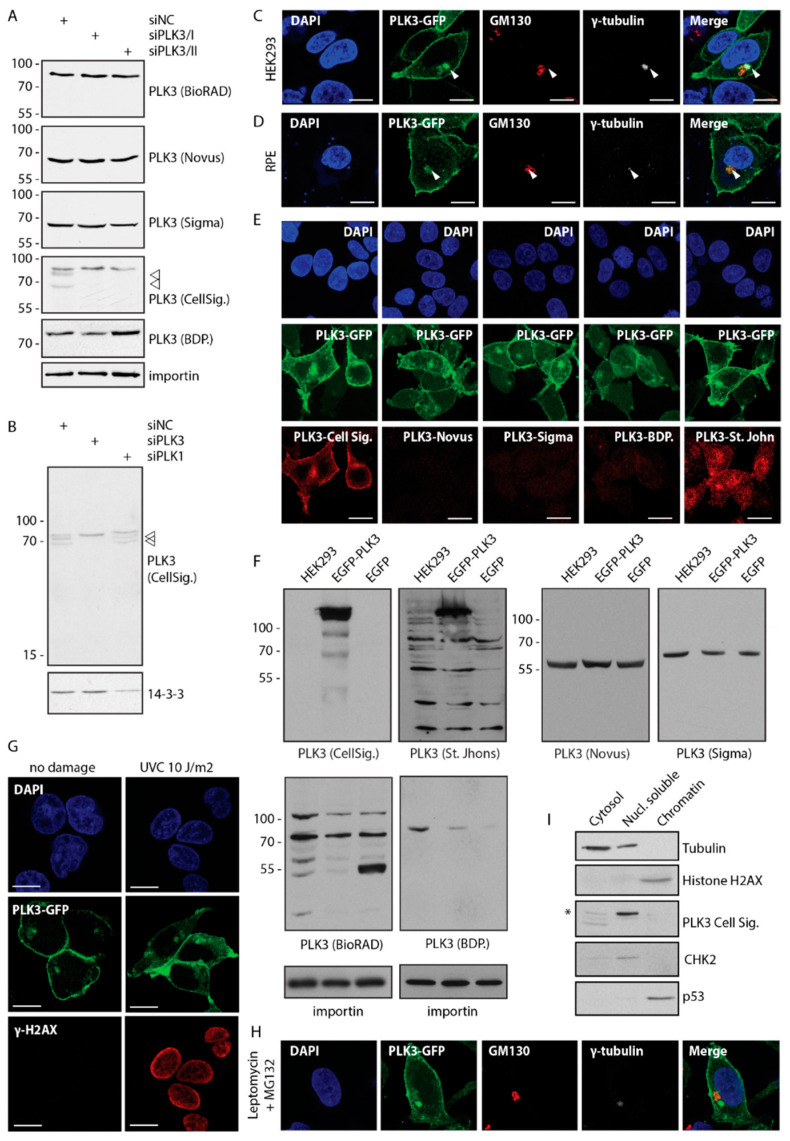
PLK3 localizes at plasma membrane and Golgi apparatus. (**A**) RPE cells were transfected with non-targeting control siRNA or two siRNAs targeting PLK3. After 48 h, whole cell lysates were separated by SDS-PAGE and probed with indicated antibodies. Numbers indicate mobility in kDa. Position of PLK3 is indicated by arrowheads. Importin was used as loading control. (**B**) RPE cells were transfected with non-targeting control siRNA or siRNA to PLK3 or PLK1. Whole cell lysates were probed with rabbit monoclonal PLK3 antibody (Cell Signaling). Protein 143−3− was used as loading control. The position of PLK3 is indicated by arrowheads. (**C**) HEK293 cells stably transfected with EGFP-PLK3 grown on coverslips were fixed and probed with antibodies against GM130 (Golgi marker) and γTubulin (centrosome marker) and imaged by confocal microscopy. The arrowhead indicates the position of the centrosome. A representative image is shown. Bar indicates 5 μm. (**D**) RPE cells transfected with EGFP-PLK3 were fixed and probed with antibodies against GM130 and γTubulin. The arrowhead indicates the position of the centrosome. A representative image is shown. Bar indicates 5 μm. (**E**) HEK293 cells expressing EGFP2−93 were fixed and probed with indicated antibodies. Bar indicates 5 μm. (**F**) Cell lysates from HEK293 and HEK293 transfected with EGFP-PLK3 or EGFP were probed with indicated antibodies by immunoblotting. Note that only PLK3 antibodies from Cell Signaling and St. Johns recognize overexpressed EGFP-PLK3. Antibody from St. Johns also shows a large number of cross-reacting bands. (**G**) HEK293 cells stably transfected with EGFP-PLK3 were fixed 2 h after exposure to mock or to UVC and imaged using confocal microscopy. Bar indicates 5 μm. (**H**) HEK293 cells stably transfected with EGFP-PLK3 were treated with leptomycin and proteasomal inhibitor MG132 for 3 h, fixed and imaged using confocal microscopy. (**I**) Cytosolic, nuclear soluble and chromatin fractions of RPE cells were probed with indicated antibodies. Tubulin is a marker of cytosol, CHK2 of the nuclear soluble fraction and histone H2AX of chromatin. Asterisk indicates non-specific band of PLK3 antibody.

Subcellular localization of PLK3 has been reported controversially. Whereas some studies identified PLK3 at the plasma membrane and Golgi apparatus, others observed enrichment of PLK3 in the nucleus and nucleolus [7,9,16,19,23]. Here, we screened all commercially available PLK3 antibodies and tested them in immunoblotting and immunofluorescence. Using siRNA-mediated knock down of PLK3, we have found that most of the antibodies recognized major cross-reacting bands but failed to recognize endogenous PLK3 migrating on the electrophoretic gel in close proximity (Figure 1A,B). The only antibody that in our hands recognized endogenous PLK3 was the rabbit monoclonal antibody (clone D14F12) from Cell Signaling Biotechnology. This antibody specifically recognized two bands migrating at 65–75 kDa (presumably corresponding to two isoforms of PLK3) and also showed a cross-reactivity with a protein of approx. 80 kDa (Figure 1A). Both bands corresponding to PLK3 disappeared after depletion of PLK3 but not of its major homologue PLK1 (Figure 1B). As none of the tested PLK3 antibodies recognized endogenous PLK3 in immunofluorescence (data not shown), we generated HEK293 cells stably expressing EGFP-PLK3 or transiently expressed EGFP-PLK3 in RPE cells. In both cell lines, EGFP-PLK3 was strongly enriched at the plasma membrane (Figure 1C,D). In addition, EGFP-PLK3 colocalized with GM130 at the Golgi apparatus, and with γ-tubulin at the centrosome (Figure 1C,D). Staining with the rabbit monoclonal antibody from Cell Signaling showed a perfectly overlapping signal with EGFP-PLK3, suggesting that it can recognize PLK3 in immunofluorescence, but its titer may be too low for detection of endogenous protein (Figure 1E). In contrast, most other PLK3 antibodies failed to recognize overexpressed EGFP-PLK3 in immunofluorescence and immunoblotting (Figure 1E,F and data not shown). Rabbit polyclonal PLK3 antibody from St. John laboratory stained centrosomes and cell nuclei, however, the nuclear signal did not co-localize with EGFP-PLK3 and was present also in cells lacking PLK3 (Figure 1E,F and data not shown). In contrast to previous reports of nuclear localization of PLK3, we did not observe accumulation of EGFP-PLK3 in the nucleus neither in non-stressed conditions or after exposure of cells to genotoxic stress (Figure 1G) [9,19]. Similarly, we found that treatment of cells with leptomycin B did not result in accumulation of EGFP-PLK3 in the nucleus, suggesting that contrary to the murine homologue Fnk, human PLK3 is not significantly shuttling between nuclear and cytosolic compartments (Figure 1H) [35]. Fractionation of RPE cells revealed that PLK3 localizes mainly to the Triton X1−00 soluble fraction containing cytosolic proteins, whereas we did not detect PLK3 in the chromatin fraction (Figure 1I). Our observations are thus in agreement with a newly described function of membrane-associated PLK3 in FasL-mediated cell death and with earlier reports implicating PLK3 in Golgi apparatus integrity [22,36].

**Figure 2:**
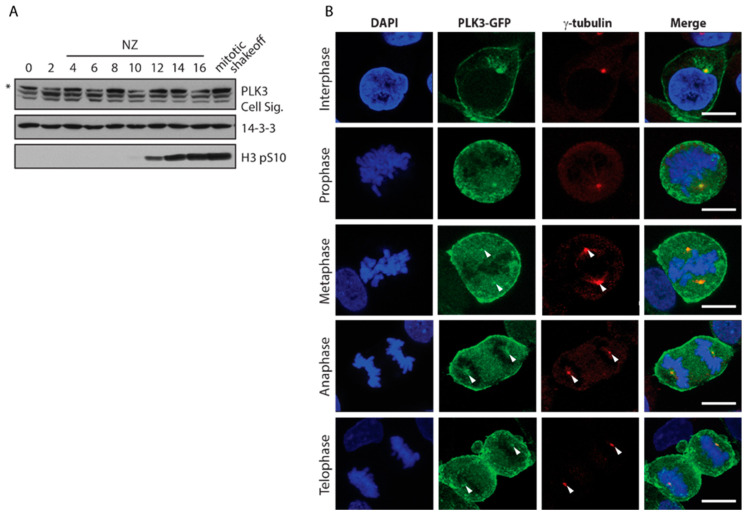
Distribution of EGFP-PLK3 during the cell cycle. (**A**) Cells were synchronized at G1/S transition with thymidine block, released to the fresh media containing nocodazole and collected in 2 h intervals. Last sample was collected by mitotic shake-off. Histone H3-pS10 is a marker of mitotic cells. Asterisk indicates non-specific reactivity. (**B**) HEK293 cells stably expressing EGFP-PLK3 were fixed, stained with an antibody against γ-tubulin, and analyzed by confocal microscopy. Maximal projections of representative cells in various phases of the cell cycle are shown. Bar indicates 10 μm.

Further, we explored the changes in EGFP-PLK3 distribution throughout the cell cycle. We found that PLK3 was expressed at comparable levels from G1 phase to mitosis (Figure 2A). Localization of PLK3 to the plasma membrane was preserved throughout mitosis (Figure 2B). In agreement with a previous report, we observed that PLK3 was also present at spindle poles during mitosis [37].

### 3.2. PLK3 is Disposable for Cell Response to Genotoxic Stress and Osmotic Stress

**Figure 3:**
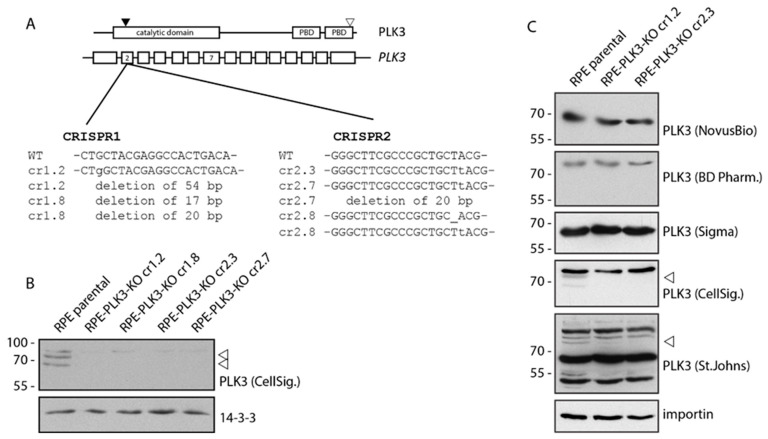
Generation of RPE-PLK3 knock-out cells. (**A**) Schematic diagram of CRISPR/Cas9-mediated targeting of human *PLK3* and of domain organization of PLK3 protein. Black arrowhead indicates the position of the critical ATP-binding Lysine 91 in the catalytic domain. Empty arrowhead indicates the position of the epitope of the PLK3 antibody from Cell Signaling. Fragments of the genomic DNA from selected clones were PCR amplified and analyzed by sequencing. (**B**) Parental RPE and selected clones of RPE-PLK3-KO cells were analyzed by immunoblotting using rabbit monoclonal PLK3 antibody (Cell Signalling). (**C**) Parental RPE and two independent clones of RP-PLK3-KO cells were probed with indicated antibodies. The position of PLK3 is indicated by an arrowhead. Please note the missing band corresponding to PLK3 in staining with antibody from St Johns laboratory and high level of cross-reactivity with other proteins.

**Figure 4:**
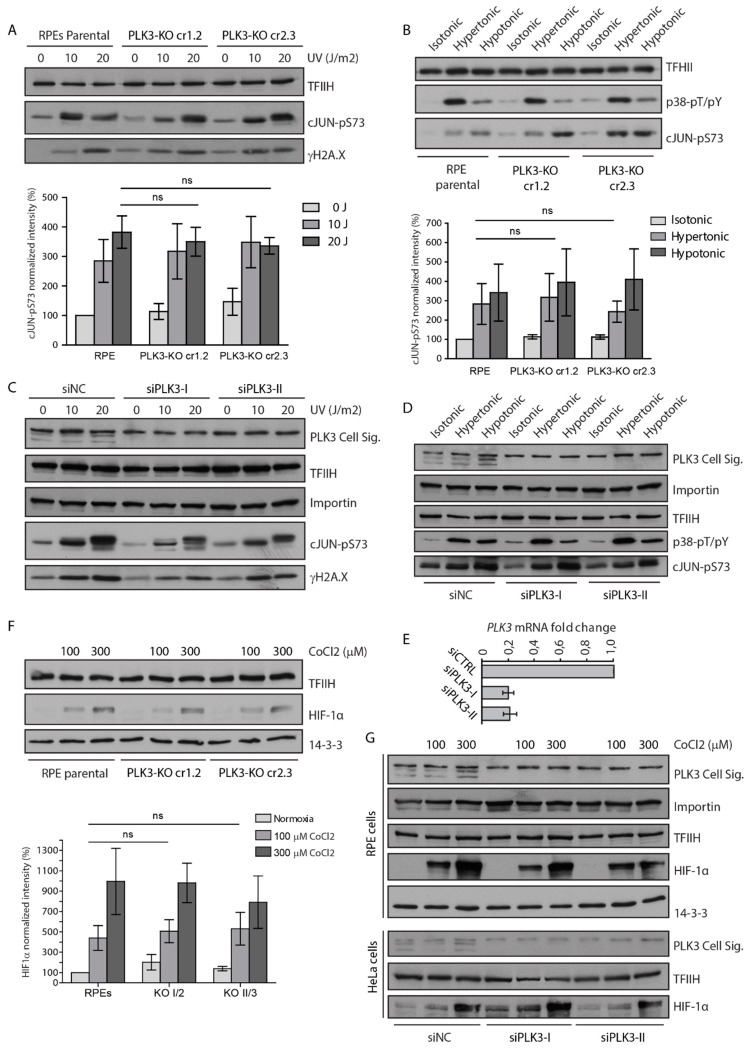

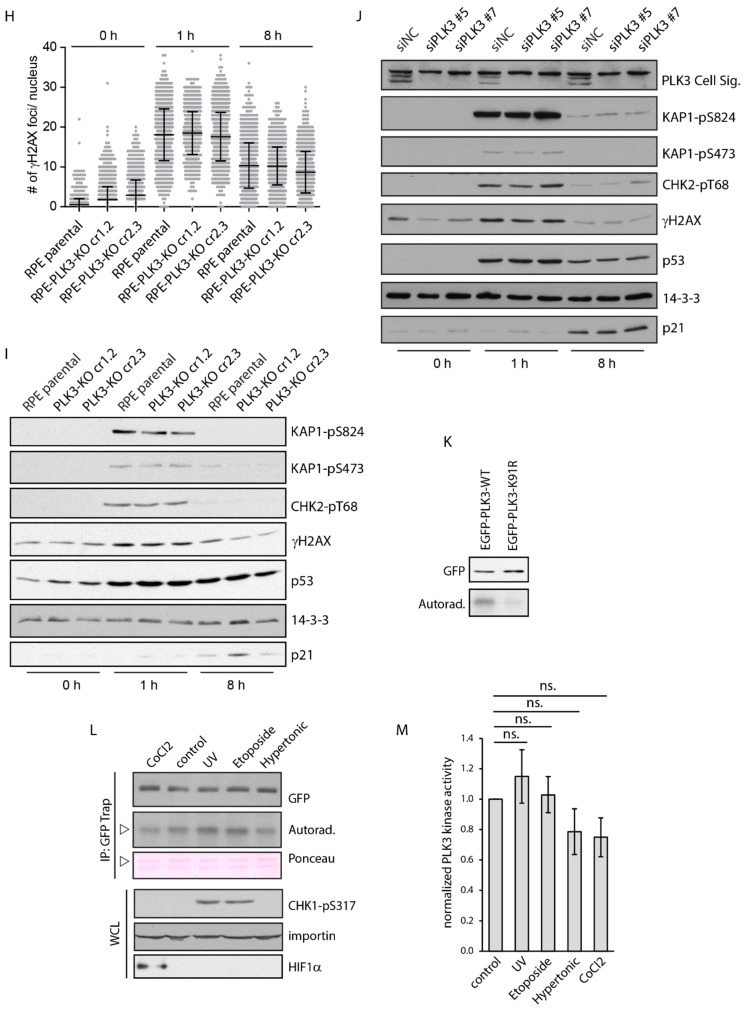
Loss of PLK3 does not affect cell response to various forms of stress. (**A**) RPE or RPE-PLK3-KO cells were exposed or not to UVC, collected after 30 min and whole cell lysates were analyzed by immunoblotting (*n* = 3). Antibody against γH2AX was used as a marker of induced DNA damage and TFIIH was used as a loading control. (**B**) RPE or RPE-PLK3-KO cells were incubated with isotonic, hypertonic or hypotonic media for 20 min and whole cell lysates were analyzed by immunoblotting. Antibody against active the form of p38-pT180/pY182 was used as a marker of cellular stress and TFIIH as loading control (*n* = 3). (**C**) RPE cells transfected with control or two various PLK3 siRNAs were treated as in A and analyzed by immunoblotting (*n* = 2). (**D**) RPE cells transfected with control or two various PLK3 siRNAs were treated as in B and analyzed by immunoblotting (*n* = 2). (**E**) Validation of PLK3 siRNA using RT-qPCR. PLK3 mRNA level was normalized to ATP5B. (**F**) RPE or RPE-PLK3-KO cells were treated or not with indicated doses of CoCl2 for 24 h and whole cell lysates were analyzed by immunoblotting (*n* = 3). (**G**) RPE and HeLa cells transfected with control or two various PLK3 siRNAs were treated or not with CoCl2 for 24 h and analyzed by immunoblotting (*n* = 2 for each cell type). (**H**) RPE or RPE-PLK3-KO cells were exposed or not to 3 Gy of ionizing radiation and fixed after 1 or 8 h. Number of γH2AX positive foci per nucleus was determined by ScanR microscopy. Lines indicate median. (**I**) RPE or RPE-PLK3-KO cells were treated as in H and whole cell lysates were analyzed by immunoblotting (*n* = 2). (**J**) RPE cells transfected with control or two various PLK3 siRNAs were treated as in H and whole cell lysates were analyzed by immunoblotting (*n* = 2). (**K**) Cells were transfected with the wild-type PLK3 or kinase-dead K91R mutant; PLK3 was isolated by GFP Trap and incubated with casein in kinase buffer supplemented with radioactive ATP. Phosphorylation of casein was detected by autoradiography. (**L**) Cells were exposed to various forms of stress including treatment with CoCl2 (300 μM, 12 h), exposure to UV (10 J/m^2^), etoposide (4 μM, 1 h), hypertonic media (480 μM mannitol) or left untreated. EGFP-PLK3 was isolated using GFP Trap and kinase assay was performed as in H. Induction of stress pathways was analyzed by immunoblotting of cell extracts. Arrowhead indicates identical position on the electrophoretic gel. (**M**) Quantification of the kinase assay in L. Signal was normalized to the non-treated control. Plotted is median +/− SD (*n* = 3). Statistical significance was evaluated by two-tail *t*-test and significance was set to *p* < 0.05. Ns. stands for non-significant.

Next, we wished to reevaluate the importance of PLK3 for various cellular functions. To this end, we knocked-out PLK3 in human diploid RPE cells using CRISPR/Cas9-mediated gene editing. We confirmed successful targeting of both alleles in the exon 2 of *PLK3* by sequencing of genomic DNA and loss of the protein expression by immunoblotting (Figure 3A–C). We exposed parental RPE and two independent clones of RPE-PLK3-KO cells to various forms of stress and probed their ability to trigger the downstream signaling. As expected, exposure of cells to UVC led to the induction of γH2AX and phosphorylation of c-Jun in parental RPE cells (Figure 4A). Surprisingly, however, we did not observe any decrease in activation of these pathways in cells lacking PLK3 (Figure 4A) [38]. Similarly, we did not observe a decreased level of the phosphorylated c-Jun after exposure of PLK3 knock-out cells to osmotic stress, suggesting that PLK3 is not involved in c-Jun phosphorylation (Figure 4B) [20]. Importantly, RNAi-mediated knock-down of PLK3 in RPE and HeLa cells also did not reveal any impact of PLK3 on the ability to activate p38 and c-Jun pathways (Figure 4C–E and data not shown). Treatment of cells with cobalt chloride mimics hypoxia and as expected, it increased levels of HIF1 alpha (Figure 4F,G) [18]. Surprisingly, we found that RPE-PLK3-KO cells or HeLa cells transfected with PLK3 siRNA induced similar levels of HIF1 alpha, suggesting that PLK3 does not inhibit HIF1 alpha stabilization in human cells (Figure 4F,G).

Finally, we evaluated the dynamics of DNA damage response by quantification of γH2AX nuclear foci formation after exposure of cells to ionizing radiation. As expected, we observed an increase in the number of nuclear foci in control cells at an early time-point after exposure to IR followed by a decrease at a later time point corresponding to DNA repair [32]. However, formation and disappearance of the γH2AX nuclear foci was comparable in parental and RPE-PLK3-KO cells (Figure 4H). Similarly, RPE-PLK3-KO cells did not show any defect in the ability to phosphorylate CHK2 at Thr68 and KAP1 at Ser824 and Ser473, suggesting that ATM and CHK2 activation is not affected in the absence of PLK3 (Figure 4I) [39,40]. Exposure of RPE-PLK3-KO cells to ionizing radiation also induced expression of p21, an established p53 target and mediator of the cell cycle checkpoint (Figure 4I). In good agreement with the data in PLK3 knock-out cells, RNAi-mediated depletion of PLK3 also did not impair the activation of DNA damage response (Figure 4J). We conclude that cells lacking PLK3 are able to activate ATM, arrest in the cell cycle checkpoint and repair DNA with comparable dynamics as parental cells.

In contrast to PLK1 that is rapidly inactivated upon genotoxic stress, PLK3 is believed to be strongly activated upon various forms of stress [12,41]. To test this, we performed an in vitro kinase assay using casein as a substrate and EGFP-PLK3 purified from cells exposed to various treatments. We found that the wild-type but not the kinase-dead PLK3-K91R mutant efficiently phosphorylated casein in vitro (Figure 4K). Surprisingly, we did not observe any significant change in the activity of PLK3 upon exposure of cells to UVC, treatment with etoposide, CoCl2, or mannitol (Figure 4L,M). These data suggest that enzymatic activity of PLK3 is not responding to stress and confirm our finding that PLK3 is disposable for cell response to genotoxic and osmotic stress.

### 3.3. PLK3 Interacts with PP6 Phosphatase Through the PBD Domain

**Figure 5:**
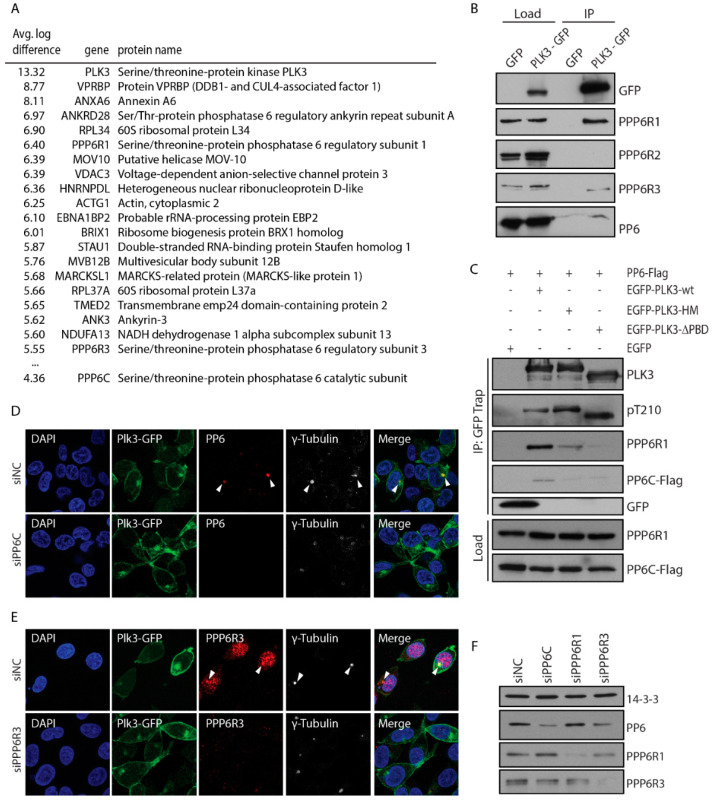
PP6 phosphatase interacts with PLK3. (**A**) Lysates from HEK293 cells expressing EGFP or EGFP-PLK3 were incubated with GFP-Trap beads, washed three times and bound proteins were analyzed by mass spectrometry. Three biological replicates were performed. Numbers indicate average log difference between EGFP-PLK3 and EGFP. Listed are top 20 hits. (**B**) EGFP or EGFP-PLK3 was immunoprecipitated from stably transfected cells, and bound proteins were analysed by immunoblotting. Representative image is shown. (**C**) Lysates from HEK293 cells transfected with EGFP-PLK3, EGFP-PLK3-∆PBD or EGFP-PLK3-H590A-K592M (referred to as PLK3-HM) mutant were incubated with GFP-Trap and bound proteins were analysed by immunoblotting. (**D**) Cells expressing EGFP-PLK3 were transfected with control siRNA or siRNA to PP6C. After 48 h, cells were fixed, stained with antibodies against PP6C and γ-Tubulin and imaged using confocal microscopy. Arrowheads indicate the position of the centrosome. (**E**) Cells expressing EGFP-PLK3 were transfected with control siRNA or siRNA to PPP6R3. After 48 h, cells were fixed, stained with antibodies against PPP6R3 and γ-Tubulin, and imaged using confocal microscopy. Arrowheads indicate the position of the centrosome. (**F**) Validation of the knock-down efficiency in D and E. Cells transfected with control siRNA or siRNA to PPP6C, PPP6R1 or PPP6R3 were lysed and probed with indicated antibodies.

To search for proteins that could modulate PLK3 function through protein-protein interaction, we immunoprecipitated EGFP-PLK3 from stable HEK293 cells and identified bound proteins using mass spectrometry. Proteins that were enriched in complex with EGFP-PLK3 compared to the EGFP in at least two out of three independent experiments were considered as potential interactors (Figure 5A). Among other proteins, we identified four components of a serine/threonine-protein phosphatase 6 highly enriched in EGFP-PLK3 complex. These included three regulatory subunits PPP6R1, PPP6R3 and ANKRD28 and a catalytic subunit PPP6C. Next, we performed immunoprecipitation from EGFP or EGFP-PLK3-expressing cells and confirmed that PLK3 specifically interacted with endogenous PPP6C, as well as with the regulatory subunits PPP6R1 and PPP6R3 (Figure 5B). In contrast, we did not observe any interaction of EGFP-PLK3 with PPP6R2 subunit.

Next we aimed to map the interaction between PLK3 and PP6. We found that the EGFP-PLK3-∆PBD mutant lacking the PBD domain showed impaired interaction with PP6 (Figure 5C). Similarly, H590A-K592M mutation of the predicted interaction site in the PBD domain also impaired interaction with PP6, suggesting that the PBD domain is needed for mediating the binding with the PP6 holoenzyme (Figure 5C) [2]. Interestingly, PP6 subunits were recently identified in a complex with PLK1, suggesting that interaction with PP6 might be conserved also for other polo-like kinase members [42]. Finally, we aimed to compare the subcellular distribution of PLK3 and PP6 complex. Immunofluorescence microscopy revealed colocalization of EGFP-PLK3 with endogenous PPP6C at the centrosome (Figure 5D,F). In addition, antibody against PPP6R3 showed a strong nuclear staining but partially also colocalized with γ-tubulin and EGFP-PLK3 at the centrosome (Figure 5E,F).

### 3.4. PLK3 Is Phosphorylated in the T-Loop but This Does Not Affect Its Kinase Activity

**Figure 6:**
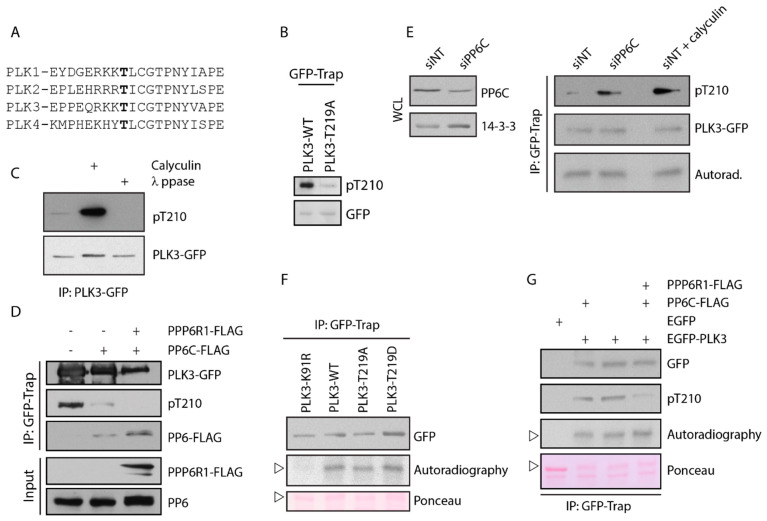
PP6 controls the phosphorylation level of PLK3. (**A**) Conservation of the T-loop of human PLK14−. Threonine residue corresponding to the T210 in PLK1 and to T219 in PLK3 is shown in bold. (**B**) EGFP-PLK3-WT and EGFP-PLK3-T219A was immunoprecipitated from transiently transfected cells using GFP-Trap and was probed with antibody against pT210-PLK1 or GFP. (**C**) EGFP-PLK3 was isolated from cells treated with mock or calyculin using GFP Trap. Isolated EGFP-PLK3 was incubated or not with lambda phosphatase for 20 min. All samples were probed with pT210 antibody. (**D**) HEK293-EGFP-PLK3 cells were transfected with PP6C and/or PPP6R1, and PLK3 was isolated using GFP-Trap. Level of PLK3 phosphorylation was evaluated by pT210 antibody. (**E**) HEK293-EGFP-PLK3 cells were transfected with negative control siRNA or siRNA to PP6C and grown for 48 h. Where indicated, cells were treated with calyculin 20 min prior to harvesting. PLK3 was isolated with GFP Trap. Level of PLK3 phosphorylation was evaluated by pT210 antibody. Kinase assay was performed as in F. (**F**) EGFP-PLK3-WT, EGFP-PLK3-T219A, EGFP-PLK3-T219D or a kinase dead EGFP-PLK3-K91R were purified from transiently transfected 293 cells using GFP Trap and incubated with casein and ^32^P-γ-ATP for 20 min at 30 °C (*n* = 2). Phosphorylation was detected by autoradiography and amount of precipitated PLK3 by staining with GFP antibody. Arrowhead indicates identical position of the casein on the electrophoretic gel. (**G**) EGFP or EGFP-PLK3-WT was purified from 293 cells transfected with mock, PP6C or PP6C with PPP6R1, and kinase assay was performed as in F. Phosphorylation of PLK3 was evaluated with pT210-PLK1 antibody and phosphorylation of the casein by autoradiography (*n* = 2). Arrowhead indicates an identical position on the gel.

A common mechanism of PLK1 activation is phosphorylation in the T-loop at Thr210 by Aurora-A kinase during G2/M transition and in mitosis [43,44,45]. As the T-loops of PLK1 and PLK3 are highly homologous, we asked if PLK3 could also be modified by phosphorylation (Figure 6A). We immunoprecipitated EGFP-PLK3 from cells and probed with an antibody directed against pT210 of PLK1 (Figure 6B). As control, we immunoprecipitated EGFP-PLK3-T219A mutant carrying a single mutation in the T-loop and confirmed that pT210-PLK1 antibody can specifically recognize phosphorylated PLK3 at Thr219 (Figure 6B). Further, we found that PLK3 was weakly phosphorylated at Thr219 in asynchronously growing cells. When we treated these cells with a broad-spectrum phosphatase inhibitor calyculin, we observed a dramatically increased level of PLK3 phosphorylation at Thr219, suggesting that modification at this site is constantly removed by an opposing phosphatase (Figure 6C) [46]. As PLK3 interacts with PP6 phosphatase, we hypothesized that phosphorylation of PLK3 could be counteracted by the activity of PP6. To test this, we isolated PLK3 from cells transiently expressing FLAG-PP6 and indeed, we found a lower level of the T-loop phosphorylation (Figure 6D). Dephosphorylation of PLK3 was further pronounced when we co-expressed PP6 with the regulatory subunit PPP6R3 (Figure 6D).

We also noted that PLK3 mutant lacking the PBD domain necessary for the interaction with PP6, showed a higher level of phosphorylation in the T-loop compared to the wild-type PLK (Figure 5C). Importantly, depletion of endogenous PP6 by RNAi increased phosphorylation of PLK3 at Thr219, indicating that PP6 phosphatase controls the level of PLK3 phosphorylation in cells (Figure 6E).

Finally, we used the in vitro kinase assay to test the contribution of the T-loop modification on PLK3 activity. We found that the wild-type PLK3 but not the kinase-dead mutant PLK3-K91R efficiently phosphorylated casein (Figure 6F). Surprisingly, however, activities of the non-phosporylable mutant PLK3-T219A and phosphomimicking mutant PLK3-T219D were comparable to the activity of the wild-type PLK3 (Figure 6F). Similarly, neither depletion of PP6 by siRNA nor the treatment of cells with calyculin affected the activity of PLK3 (Figure 6E). Finally, overexpression of PP6C together with its regulatory subunit PPP6R1 did not affect the activity of the immunoprecipitated EGFP-PLK3 (Figure 6G). We conclude that activation of PLK3 does not require phosphorylation of the T-loop at Thr219 and that PP6 phosphatase does not control the level of PLK3 activity in cells.

## 4. Discussion

In this study, we knocked-out *PLK3* in human RPE cells using CRISPR/Cas9-mediated gene editing to study PLK3 function in stress response. Two independent clones of RPE-PLK3-KO showed no differences in stabilization of HIF1 in hypoxia conditions, in phosphorylation of c-Jun after exposure of cells to UV irradiation and in activation of DNA damage response after exposure of cells to ionizing radiation. Importantly, we obtained similar results when we depleted PLK3 using RNAi in human diploid RPE cells or in transformed HeLa and U2OS cells. Our data suggest that PLK3 does not significantly contribute to the cell response to hypoxia or DNA damage in human cells. In agreement with this, kinase assays performed using EGFP-PLK3 purified from cells exposed or not to various forms of stress also did not show any significant increase in the kinase activity of PLK3. Specificity of the kinase assay used in this study was validated by a kinase-dead mutant EGFP-PLK3-K91R that did not show any enzymatic activity. We conclude that in human cells, PLK3 does not respond to cellular stress. Previous reports relied mostly on purification of endogenous PLK3 using a polyclonal antibody, the specificity of which was not validated [16,19,20]. Since this antibody is no longer available, we could not perform the kinase assays in parallel with our assay. We tested several other available antibodies but most of them did not show satisfactory specificity and sensitivity towards PLK3. The only antibody that in our hands reliably recognized endogenous PLK3 in immunoblotting was a rabbit monoclonal from the Cell Signaling, but the antibody was not suitable for immunofluorescence microscopy. Other antibodies demonstrated poor affinity to exogenously expressed PLK3 and showed strong cross-reactivity in immunoblotting and a non-specific nuclear staining in immunofluorescence. We believe that previous data implicating PLK3 in the stress response in human cells should be interpreted with caution as they could be affected by low specificity of primary antibodies to PLK3. In this study, we observed enrichment of EGFP-PLK3 at the plasma membrane, which is in agreement with the recently described function of PLK3 in Fas ligand-induced apoptosis [36]. However, we were unable to validate this novel function of PLK3 because RPE cells are resistant to FasL treatment (data not shown).

Further, we show that PLK3 is post-translationally modified at Thr219 within the T-loop. The level of this phosphorylation is regulated by the PP6 holoenzyme that interacts with and continuously dephosphorylates PLK3. PP6 has recently been shown to regulate the activity of ASK3 kinase by controlling its phosphorylation upon osmotic stress conditions [47]. Therefore, we tested if PP6 could control PLK3 activity. However, we did not observe any changes in PLK3 interaction with PP6 upon osmotic stress (data not shown) and also activity of PLK3 was not affected by depletion of PP6, overexpression of PP6 or by treatment of cells with the phosphatase inhibitor calyculin. Phosphomimicking T219D and non-phosphorylatable T219A PLK3 mutants showed comparable enzymatic activities as the wild-type PLK3, suggesting that PLK3 is regulated by more distinct mechanisms than PLK1. It is possible that a single modification of the T-loop of PLK3 is not sufficient to boost the kinase activity of PLK3 and additional modifications may exist that control its function in cells. Instead of being regulated by PP6 through the T-loop modification, PLK3 could also stand upstream of the PP6 holoenzyme and control its function by targeting some of its subunits, which remains to be addressed by future research. We did not observe any changes in the level of phosphorylated Aurora-A at T288 in RPE-PLK3-KO cells, suggesting that PLK3 does not affect the PP6-Aurora-A axis during mitosis as has been reported for PLK1 (data not shown) [27,42]. Given the enrichment of PLK3 at the plasma membrane and Golgi apparatus, it will be interesting to test its potential impact on cell adhesion and intracellular trafficking that are regulated by PP6 phosphatase [48,49]. Finally, there is emerging evidence that expression of PLK3 could affect the therapeutical response in melanoma, prostate cancer and colon carcinoma [50,51,52]. Further phosphoproteomic studies are needed to identify new substrates of PLK3 that could explain its role in cell physiology and sensitivity to chemotherapy.
